# Reconsidering the role of place in health and welfare services: lessons from the COVID-19 pandemic in the United States and Canada

**DOI:** 10.1007/s42532-022-00111-z

**Published:** 2022-04-19

**Authors:** G. Allen Ratliff, Cindy A. Sousa, Genevieve Graaf, Bree Akesson, Susan P. Kemp

**Affiliations:** 1 Miami University, Oxford, OH USA; 2 Bryn Mawr College, Bryn Mawr, PA USA; 3 University of Texas at Arlington, Arlington, TX USA; 4 Wilfrid Laurier University, Ontario, Canada; 5 University of Auckland, Auckland, New Zealand

**Keywords:** Place, Place theory, COVID-19 pandemic, Wellbeing, Social welfare, Health

## Abstract

Places—the meaningful locations of daily life—have been central to the wellbeing of humans since they first formed social groups, providing a stable base for individuals, families, and communities. In the United States and Canada, as elsewhere, place also plays a foundational role in the provision of critical social and health services and resources. Yet the globally destabilizing events of the COVID-19 pandemic have dramatically challenged the concept, experience, and meaning of place. Place-centered public health measures such as lockdowns and stay-at-home orders have disrupted and transformed homes, neighborhoods, workplaces, and schools. These measures stressed families and communities, particularly among marginalized groups, and made the delivery of vital resources and services more difficult. At the same time, the pandemic has stimulated a range of creative and resilient responses. Building from an overview of these effects and drawing conceptually on theories of people–place relationships, this paper argues for critical attention to reconsidering and re-envisioning prevailing assumptions about place-centric policies, services, and practices. Such reappraisal is vital to ensuring that, going forward, scholars, policymakers, and practitioners can effectively design and deliver services capable of maintaining social connections, safety, and wellbeing in contexts of uncertainty, inequality, and flux.

## The places of COVID-19

In the United States and Canada, as in many other countries, the COVID-19 pandemic and subsequent public health responses—such as stay-at-home orders and social distancing mandates—have profoundly complicated the concept, experience, and meaning of “place.” Policies and practices aimed at controlling the pandemic have turned homes into workplaces, childcare centers, and schools, contributing to stress, heightened incidence of family violence, and, for many, even greater overcrowding. Homes have also become places of social isolation and neglect as pandemic controls have disrupted or reduced the home-based care depended upon by older adults and individuals with disabilities or complex medical needs. Stay-at-home orders have likewise transformed everyday community connections and resources, emptying neighborhoods, isolating households, and constricting social interactions within places we normally rely on for social support and connection, such as schools, libraries, restaurants, and community centers.

The detrimental effects of the pandemic on places as sites of health, safety, and service provision have fallen most heavily on marginalized and minoritized populations, deepening pre-existing vulnerabilities resulting from generations of racism, economic disenfranchisement, food insecurity, inadequate housing, and inaccessible healthcare (Almagro et al. 2021; Bowleg 2020). For example, in large cities in the United States and Canada, essential workers such as farm and factory workers, caregivers for older adults and disabled people, public transportation operators, and supermarket and food service workers are disproportionately people of color and immigrants who already face structural inequities related to economic precarity, residential crowding, and racism (Almagro et al. 2021).

The effects of the pandemic on people’s experiences with everyday places have been widely felt and discussed. Less examined have been the place-related implications of pandemic responses in terms of social policies, services, and systems. At the national, state, and local levels, health and welfare policies and services rely centrally on assumptions that place is both a relatively stable and accessible site of intervention, and, in general, a locus of safety and wellbeing. The COVID-19 pandemic, however, has upended many of these taken-for-granted assumptions. Place-based services—home- and community-based care, community centers, and other localized services—have been disrupted by lockdowns, limiting access to healthcare, childcare, employment, and supportive resources (Shang et al. 2020). The multiplying stressors associated with stay-at-home orders, quarantines, and illness have likewise challenged assumptions about homes as havens. For those already unstably housed or homeless, for example, the pandemic has deepened their precarity, precipitating evictions and adding additional health and mental risks.

The current moment thus brings squarely into view the need for critical re-consideration of prevailing assumptions about the positioning and the role of “place” in the design and delivery of resources and services necessary for people to meet basic needs and safeguard their wellbeing. Revisiting these previously taken-for-granted assumptions is particularly important given the increasing need for social welfare and public health policies and services capable of maintaining social connections, safety, and wellbeing in contexts of uncertainty and flux.

To support our arguments for such re-consideration, we first look broadly at place-related responses to the COVID-19 pandemic, grounding these conceptually in a brief discussion of key dimensions of the relationships between places, people, and wellbeing. We then look in more detail at the place-related impacts of the COVID-19 pandemic on people’s daily lives, focusing on places such as homes, neighborhoods, workplaces, and schools that are typically assumed to be stable and relatively coherent. After examining related challenges in providing human services to those in need amid the pandemic, we conclude by exploring the conceptual and practical implications of the place-related impacts of COVID-19 for social policies, services, and supports. While these dynamics are clearly global in scope, we focus this paper primarily on the United States and Canada.

### Considering COVID-19 and place

The spatial constraints associated with the COVID-19 pandemic have complicated and transformed conventional understandings of places and people’s relationships with them, revealing the complexities inherent in notions of place and its role in human lives. In early 2020, COVID-19 evolved from a minor news story to an alarming global concern. The World Health Organization (WHO) initially recommended rapid testing and containment to help avoid community spread (WHO 2020), with many cities containing active cases in quarantine centers (WHO 2020). When it became apparent that cases were rapidly outpacing localized quarantine strategies, primary recommendations turned to social distancing and quarantining within homes (WHO 2021). As the pandemic expanded, containment strategies progressed toward recommendations that entire populations isolate, subsequently closing schools, workplaces, and borders under collective stay-at-home orders. In much of the world, stay-at-home orders, initially projected by the Centers for Disease Control and Prevention (CDC) to only last for weeks, were subsequently extended to months and years (CDC 2020).

These public health measures have forced people to isolate and in some cases suffer in place, enduring mental and physical health concerns (including but not only COVID-19), the strains of child and elder care, and family strife without physical access to the professional external supports they might normally use. The restrictions on access to places, and emphases on containment at the heart of pandemic responses have also brought to the foreground the many ways—both practically and philosophically—people connect personal freedom and wellbeing with ready access to place and space (Tuan 1977). Previously, public health interventions that curtailed movement and free use of place were novel and rarely used strategies in the United States and Canada, where the right to access and use public spaces has typically been taken for granted. Faced with significant constraints, people and services have therefore been forced to both innovate and improvise. Through creativity, determination, and necessity, the demands of the pandemic have thus also stimulated fresh thinking about place and its functions.

In following sections, we describe the implications of the unprecedented spatial interventions of the pandemic in more detail. As a starting point, however, the concept of place itself requires further elaboration.

## Place as location, meaning, and power

Place is a tricky concept to define, as it is inherently multidimensional, spanning physical, subjective, and social environments, at multiple levels from personal spaces to macro-level social structures (Kemp 2010). Three main dimensions of place are however particularly relevant to our focus on place in the context of the COVID pandemic. First, place as a physical location where resources are obtained and distributed. Second, place as a site of meaning and identity—a setting people identify with and within which they connect with others and with personal, social, and cultural resources. Third, place as constructed and maintained through power dynamics. Adding to these, scholars of place have identified three features of people–place relationships of particular relevance in the context of the pandemic, specifically the dialectics of inclusion–exclusion, inside-outside, and fixity-flow (Devine-Wright et al. 2020).

### Place as a physical location

Notions of place and home operate across geographic scales, from family dwellings and office buildings to neighborhoods, cities, regions, states, and nations (Blunt and Dowling 2006). No matter the scale, the most obvious dimension of place is as a real-world physical location: places exist somewhere, can be found on a map, and typically contain the resources we need to survive. For example, a home is generally a location where we live, make daily use of meaningful and useful everyday objects, host other people, and build personal and relational connections with people and the surrounding environment (Young 2005). During the COVID-19 pandemic, the physicality of everyday places grew both more tangible (given spatial constraints) and more abstract, as places like classrooms, workspaces, and health clinics shifted from physical to virtual locations. In many cases, people were at once located physically within their homes and virtually in other “places” of their day-to-day lives. This hybridization of place experiences allowed us to maintain essential connections with others from whom we were isolated, whether as close as next door or as far away as the other side of the world. Yet it also disrupted physical contact with other people and experiences, illustrating how constraints on place prevent the spontaneous, organic connections upon which the development and maintenance of community rests.

### Place as attachment, identity, and wellbeing

Relationships to “strong, well-developed, nurturing places” provide feelings of attachment and belonging that support psychological and physical wellbeing (Fullilove 1996, p. 1517; Fullilove et al. 1998). Place attachment also nurtures place identity—our sense of ourselves as a function of our relationships with particular places and the people in those places (Low and Altman 1992). We experience place identity both as individuals and as a collective (Low and Altman 1992; Proshansky et al. 1983). In the pandemic, the need to isolate in homes and monitor interactions with locations we normally access freely (e.g., schools, workplaces, grocery stores, public and commercial centers, the houses of friends and family) has interrupted routines typically at the heart of place identity and belonging, contributing further to individual and family and collective stress (Czeisler 2021; Czeisler et al. 2020; Ettman et al. 2020).

### Place and power

In addition to their physical location and social meaning, places are inherently sites of power. Everyday places are physically and socially constructed, altered, and maintained by systems of power as well as by the needs and decisions of the people interacting with those places. A critical element of the relationship between place and wellbeing is the amount of power we have over our places. Seemingly mundane experiences within place reflect important relationships to and negotiations with larger power structures (Kemp 2010). The COVID-19 pandemic has sharply highlighted how power works in and through places, illustrated by decisions regarding who can move into and between places, when and how individuals can access certain places, and how places may operate. In the United States, for example, restrictive policies to control viral spread have varied by city, county, and state (Wang and Pagán 2021), resulting in widely differing experiences of freedom and safety for residents.

### Place dialectics

Three place dialectics—emplacement-displacement, inside-outside, and fixity-flow—call attention to the complexities and dynamics inherent in places and place experiences in the context of a global pandemic (Devine-Wright et al. 2020). Emplacement–displacement captures the phenomenon of being simultaneously contained in some places (as happened with stay-at-home orders during the pandemic) and displaced from others—as seen in the ways that social distancing and stay-at-home orders prevented people from accessing public and private spaces that were closed or deemed unsafe, or from traveling to see loved ones who live in other places. The construct ‘inside-outside’ illuminates similarly dialectical processes—the reality that those who are allowed inside given places (whether a home or a country) are at the same time connected to what is occurring elsewhere. Prior to the pandemic, although a range of policies defined who could traverse various thresholds (such as national borders), most individuals retained a reasonably high degree of freedom of movement. During the pandemic, travel restrictions, border controls, quarantine “bubbles,” and “window visits” with loved ones in congregate care settings have both highlighted and disrupted the complex balance between insideness and outsideness typical of people’s everyday lives. The third construct, ‘fixity-flow’ captures the tensions and negotiations inherent in the ways that people balance locatedness in place and movement across places—patterns of subjectivity, habit, and agency that the constraints of the pandemic have likewise disrupted. Taken together, these dialectics offer helpful perspectives on the “inherent contradictions and active (re)negotiation of place experience in which people must now engage” (Devine-Wright et al. 2020, p. 3).

## Place-based impacts of COVID-19

COVID-19 has brought into sharp relief the ways in which the physical dimensions of place intersect with power structures to perpetuate and deepen disproportional impacts on the wellbeing of people in particular areas and places. To explore these interacting dynamics, we begin at the center of private life, the home, to consider the ways that COVID-19 has complicated the meaning of home in people’s daily lives. Zooming out, we then consider how the pandemic has shifted understandings and experiences of places of public life, including neighborhoods, workplaces, and schools. The impacts of the pandemic have at once contributed to a greater need for the services typically tied to these places and limited access to them. In the context of heightened risk, there have also been instances of notable creativity. This dimensionality underscores the importance of attention to the complex interactions among place, health, and wellbeing, and related responses.

### Home as “castle and cage”

Scholarship on “home” and its implications is inherently dualistic. Place scholars have described “at-home-ness” as the unique sense of comfort and safety that people derive from their domestic spaces (Seamon 1979, p. 70). At the same time, feminist and other critical scholars have exposed the home as a site of oppression, highlighting the endless drudgery of housework and the violence that women and children may experience in the home (Manzo 2003). Scholarship exploring these co-existing realities has included research on the meaning of home for people who were victims of domestic or political violence (Meth 2003; Sousa et al. 2014); unhoused people with mental illnesses (Padgett 2007); and children facing adversities, including war and disabilities (Akesson 2014; Yantzi and Rosenberg 2008). This body of work highlights the complex ways we construct and experience home places, illustrating the dynamic nature of places we seek refuge within but may also experience as sites of discomfort, fear, relentless caretaking, or isolation: the reality, indeed, of home as simultaneously “castle and cage” (Akesson 2014).

The pandemic has underscored these insights. Even in less-crowded conditions, privacy and ease—critical emotional and practical dimensions of home (Young 2005)—could not be taken for granted in the context of stay-at-home orders. In crowded and impoverished housing conditions, the impacts of these have been significantly more dire (Hu et al. 2021). For example, for those living in multi-unit housing in the United States (disproportionately non-white populations), the economic and structural constraints of inadequate housing resulted in higher risk of disease exposure. Shared entries, laundry facilities, narrow hallways, and communal mail pick-up contributed to increased risk of exposure to COVID-19 when people were attempting to fulfill daily tasks (Williams and Cooper 2020). Meanwhile, the open spaces (e.g., parks) that people might use to alleviate isolation and crowding are not equitably accessible to marginalized groups, further limiting options for sustaining physical and mental health amid the pandemic (Hoover and Lim 2021).

For people in all strata of society, containment at home with little outside assistance exacerbated gender inequities, as caretaking responsibilities, which disproportionately fell on women, consumed even more time and energy (Yavorsky et al. 2021). As schools and family care centers closed, home-based caretakers had to step in to meet the multiple practical and emotional needs of family members. Furthermore, as people were isolated in their homes and displaced from the public spaces of their neighborhoods and cities, an array of typically external activities (e.g., work, school, socializing, exercise, mourning) moved into the domestic space. Without breaks or access to quiet, restorative spaces and practices, these additional responsibilities have led to increased exhaustion and mental health issues (Almeida et al. 2020; Thibaut and van Wijngaarden-Cremers 2020).

For adults and children experiencing abuse in the home, orders that forced them to stay at home posed considerable additional risks, as violence increased and lockdowns and quarantines compromised possibilities for escape (Boserup et al. 2020; Herrenkohl et al. 2021). Pandemic-related stressors, both individual-level (insecurity, confusion, isolation, and stigma) and community-level (economic loss, work and school closures, inadequate medical resources, scarcity of food and other necessities), exacerbated substance use and mental health conditions (Czeisler et al. 2020; Ettman et al. 2020).

Findings on the impacts of containment measures in terms of everyday hassles, physical and mental health crises, escalating violence, and diminished social support affirm prior scholarship questioning assumptions that homes are a one-dimensional source of safety, comfort, and ease (Mallett 2004). Yet scholars have also pointed to the protective effects of being anchored to home during the pandemic (Ahrens et al. 2021; Shoshani and Kor 2021). Even (or especially) amid a global crisis, homes still offer peace, comfort, recreation, learning, social support, communication, and positive identity formation (Ahrens et al. 2021; Panchal et al. 2021; Shoshani and Kor 2021). The pandemic has thus not entirely shifted our understandings of home. Rather, it has brought into sharp relief the complex and dynamic relationships between wellness and stress, belonging and isolation, ease and threat, that homes represent.

### Neighborhoods

Neighborhoods are geographically small, bounded, symbolically influential spaces that are meaningful to residents and remain relatively stable over time. Sinha (2006) describes neighborhoods as a “shared locality [that] gives rise to strong sentimental bonds between residents who are linked through neighborhood-based systems of activity and organization” (pp. 14–15). The stability and social bonds offered by neighborhoods have been both challenged and in some ways invigorated by the pandemic. Research in several different communities in the United States and Canada, for example, demonstrates that at the neighborhood level, people have felt isolated within their homes yet nervous when approached by neighbors outside the home (Bateman et al. 2021; Herron et al. 2021), increasing fear, social insecurity, and social isolation (Zetterberg et al. 2021). For many people, the pandemic has disrupted the daily activities and routines that build connections in the context of proximity, weakening social bonds between neighbors and underscoring the need for social distancing. At the same time, the pandemic has also seen a flourishing of neighborhood-level outreach, caretaking and mutual aid, as community members have sought to bridge gaps in resources and services and take care of vulnerable community members (Bell 2021; Lofton et al. 2022).

High-density neighborhoods present particular complexities. In these settings, the pandemic highlights both the risks of population density related to disease transmission (Carrión et al. 2020; Sy et al. 2020) and the potential benefits of this density regarding increased availability of services, such as healthcare and the delivery of food and other items. One major study of more than 900 urban centers in the United States found that while there was a relationship between higher population density and increased infection, the relationship dissipates with intervening factors such as access to healthcare and maintenance of social distancing (Hamidi et al. 2020). In one qualitative study, participants suggested that it was difficult to enact social distancing in crowded housing communities (Bateman et al. 2021). Studies describe neighborhood housing quality and social disadvantage as pathways to increased COVID-19 infections, specifically via the inability of residents in high-density housing situations to adequately social distance and quarantine to prevent viral spread (Carrión et al. 2020). In addition, many residents in poor, socially disadvantaged communities are more likely to be considered essential workers in low-status professions such as delivery, factory, and supermarket workers. These employees have been at the forefront of viral transmission, infection, and fatalities, further deepening health risks in neighborhoods already at high risk due to population density and poor access to healthcare and services (Cole et al. 2020).

### Workplaces

Before the COVID-19 pandemic, most people worked outside the home, clearly separating work and home life (Laing 1991). Of the 38% of the workforce reporting the option to work from home, only 20% of those reported working from home all or most of the time (Parker et al. 2020). Many organizations were slowly moving to a more “flexible” workspace to better accommodate the needs of workers and reduce overhead costs (Harris 2015; Jeffrey Hill et al. 2008). The pandemic spurred employers to enact and support remote work formats.

Research on the experiences of those working from home during the pandemic presents a mixed picture. Many of those able to work remotely via video conferencing and Internet-based technologies experienced increased strain from merging workspaces and living spaces. Home-based work also introduced challenges in obtaining the technological resources to work comfortably and effectively, and in sustaining motivation given interruptions to work productivity due to family care or educational support for children (Parker et al. 2020).

Although mixing home and workplaces brought complexities—especially when schools or childcare options were less available to working parents—employees who could move their work into their home also experienced benefits. Those working at home reported increased work-life balance, work efficiency, and control over their workday (Ipsen et al. 2021). A survey of Canadians who worked from home during the pandemic found that two-thirds expected to continue remote work once the pandemic had ended (Kurl and Korzinski 2020).

However, many essential workers—not only medical personnel, but also grocery workers, teachers, cleaning professionals, transportation workers, and farmworkers, among others—were not offered the possibility of choice with regard to their location of work or even assured of any safety procedures to protect themselves (Béland et al. 2020). In the United States, workers with lower wages, lower education, and who identify as Latinx and African American disproportionately occupy these positions (Maness et al. 2021; McClure et al. 2020; St-Denis 2020). Demonstrating the complex interactions between place and power during the COVID-19 pandemic, in many of these contexts, economic interests have dictated the risks associated with where and how individuals work, earn, and live. The pandemic has illuminated the need for policies that mitigate these risks, such as affordable housing for migrant workers, who often live together in employer-provided housing, drive to work together, and work in close quarters, making social distancing precautions all but impossible and putting them at particular risk for contracting COVID-19 (Lay 2020).

### Schools

Children and youth spend most of their time away from home in school. Schools are places that educate and socialize children and where many of those with social, economic, physical, or behavioral challenges obtain critical supports and services. In school settings, children and young people also form important peer friendships and relationships with adult mentors, receive essential social, nutritional, and health care resources, and access behavioral or educational supports (Brener et al. 2007; Helseth and Frazier 2018).

In March 2020, schools across the United States closed their doors, scrambling to move learning to online platforms and still provide critically important school meal programs to millions of children in low-income households (Dunn et al. 2020). These changes placed children at increased risk of family violence (Usher et al. 2020). With fewer mandated reporters observing the physical or emotional wellbeing of children, reports of child abuse and neglect dropped sharply in the months following school shut-downs, alarming child welfare researchers and administrators (Herrenkohl et al. 2021; Masonbrink and Hurley 2020). Children with special needs suffered from decreased or eliminated provision of critical educational accommodations or supports (Grooms and Childs 2021), while caregivers’ stress and mental health concerns increased as they struggled to fill gaps left by the loss of formal supports (Chan and Fung 2021). All children suffered from lack of social interaction and decreased physical activity (Racine et al. 2020), resulting in spikes in child psychiatric emergency room visits (Krass et al. 2021) and rates of childhood obesity (Browne et al. 2021). However, children in lower-income families experienced greater educational impacts due to reduced access to high-speed Internet and appropriate technology, and (with many caregivers working outside the home) a lack of adult support or supervision for online schooling (Domina et al. 2021; Dorn et al. 2020; Kraft et al. 2020). These disproportionate educational impacts may contribute to continuing economic disparities resulting from the pandemic, making it harder for affected groups to recover in years to come (Snowden and Graaf 2021).

### Health and human services

The shrinking parameters of home, school, and work in the context of the pandemic have created challenges in both providing and accessing needed services and resources. In this context, the extent to which existing health and welfare interventions rely on place-based services has become increasingly apparent. Policies restricting mobility have destabilized social and human services, limiting access to mental and physical healthcare, childcare and employment, and safe and supportive resources. Closure of childcare centers has disproportionately impacted women, as mandates forced parents to stay home with young children, a task that disproportionally impacts mothers (Alon et al. 2020). Providers of physical or occupational therapy, personal care services (e.g., assistance with hygiene or grooming, meal preparation, toileting), and paraprofessional behavioral support continued to provide in-person services, often at increased risk to both service recipients and service providers (Guerrero et al. 2020; Shang et al. 2020). When service supply was insufficient for public need, or when risk was deemed too high, family members often provided this care (Chan et al. 2020; Phillips et al. 2020), increasing individual and family stress and caregiver-burnout (Czeisler 2021; Greenberg et al. 2020).

Many health and social services shifted to virtual or hybrid formats for service delivery (Carlo et al. 2021; Font 2021), raising questions about effectiveness and equity. Where possible, individuals and families accessed virtual support, such as health and mental health care services through telehealth formats. Yet the accessibility of such services is dependent on multiple factors, including knowing who to contact for help, having a reliable Internet connection and sufficient data access, having the ability to speak freely and privately about one’s experiences and needs within one’s current setting, and the extent to which virtual services are regarded as culturally safe, or not. All of these factors raise the likelihood that extant disparities in access to and provision of needed services will be deepened further, even though virtual services may have other benefits.

Other place-based service settings were also extremely challenged by the pandemic. In the United States, residents in congregate settings (e.g., jails, prisons, residential group homes, and nursing homes), who are disproportionately non-white, faced additional risk of exposure to COVID-19. These communal places became hotspots for the spread of COVID-19 (Barnett and Grabowski 2020). Due to challenges in managing spread unique to congregate settings (Rubin 2020; Shippee et al. 2020), both residents and workers were at risk of becoming ill and dying at greater rates than those in non-congregate settings, further stretching already over-burdened services.

Across these varied contexts, COVID-19 has brought to the fore the centrality of place in disparities in health outcomes, risks, and access to resources and services. At the same time, it has raised awareness of the importance of homes and local places in human safety and security, and stimulated a range of creative and perhaps durable place-centered responses. In what follows, we explore the implications of this dual reality for social relationships, direct services, and the realignment of policies going forward, focusing on areas where responses to the pandemic open fresh perspectives on place and its role in sustaining or undermining wellbeing. We do so in the spirit of critical place inquiry, which confronts “critical questions…informed by the embeddedness of social life in and with places, and… seeks to be a form of action in responding to critical place issues” (Tuck and McKenzie 2015, p. 2).

## Re-imagining place in health and human services and policies

The COVID pandemic fundamentally disrupted people’s relationships with all four of the everyday places we have reviewed. Homes that for many were sites of safety and sanctuary from the outside world frequently took on additional meanings as locations of amplified burden, strife, and role confusion. Neighborhoods that were places of bonding, economic liveliness, and practical resources temporarily became sites of isolation and silence. Workplaces and schools disappeared into homes or became vectors of disease risk.

Before the pandemic, many people’s lives were increasingly globally connected, mobile, and virtually mediated. Although people still lived in physical spaces, and most social and health policies and services remained predicated on understandings of place as a particular physical location, larger trends evidenced shifts toward experiences and opportunities unanchored in geography. In complex ways, the pandemic upended this fluidity. It has both fixed people in place (particularly in the home), and greatly expanded the use of virtual spaces as locations for work, education, social support, and health care. These shifts have unsettled individuals, families, and communities, and destabilized extant services, policy, and planning assumptions.

As the world begins to look ahead to a post-pandemic “new normal,” what are the potential lessons of the pandemic for place-centric health and human services and policies? Two domains seem particularly salient: a re-emphasis on local capacity, connections, and resources, and a re-envisioning of place to include virtual spaces.

### Reasserting the local

Research has provided hints that, despite the hollowing out of street life that happened at the height of the pandemic, COVID-19 also inspired creative responses in neighborhoods and communities. For example, as mitigation strategies constricted people’s scope of travel, they were compelled to find meaning and connection close to home (Gatti and Procentese 2021). Without access to broader networks and sets of resources, the local became more critical. People checked on each other, resisted sweeps of encampments for the unhoused, made, shared, and wore masks in social solidarity (Cheng et al. 2020), shared resources to care for children, and re-created rituals to build local community. As it became more apparent that risks from outdoor transmission were relatively low (mainly from research conducted after racial justice protests in the summer of 2020), ordinary places, including streets, were reclaimed as essential gathering places—helping people and businesses to nurture community and connection. This re-engagement with place illustrates the enduring relevance of place attachment and place identity and their salience in individual and collective wellbeing.

The exigencies of the pandemic have also stimulated fresh approaches to place-centered health and human services, often involving the use of both novel and nimble delivery mechanisms aimed at getting services as close to people in their local contexts as possible. Examples include the use of mobile clinics to improve COVID-19 vaccination and testing outreach, or of food trucks and community food fridges (Lofton et al. 2022) to provide access to food in areas facing scarcity.

Sustaining these efforts going forward, many of which have reached those least well-served by present services, will require continuing recognition of the importance of neighborhood-based care, together with advocacy for policy-level and funding investment to support locally responsive, equity-oriented services. Building from the lessons of the COVID-19 pandemic, cities aiming to mitigate the effects of future pandemics or other disasters have the opportunity to invest in strengthening community caretaking and mutual aid, for example by supporting grassroots, locally accessible services and organizations. Neighborhood associations and community centers can likewise partner with local governments to advocate for increased accessibility to Internet services and affordable or free computers, and for funding aimed at supporting local community-building and outreach activities.

Collaborations between novel community partners, such as health providers and educators, became essential to bridging gaps in service delivery created, highlighted, or exacerbated by the pandemic (Jablonski et al. 2021; Tremmel et al. 2020). The opportunity exists to maintain many of these partnerships well beyond the resolution of the current health crisis. The pandemic has also opened vistas on the potential for better connecting otherwise siloed services, such as building collaborations between schools, healthcare, social services, and food distribution that enable families in need to flexibly and easily access vital resources.

### Re-envisioning the virtual

The pandemic has also required policymakers and service providers to envision and make significantly more use of “place” in virtual terms—a conceptualization that has not previously been at the center of public and policy discourse. The constraints of the pandemic have underscored the importance of virtual as well as physical access to a wide range of resources. Despite challenges, the online options that have proliferated in education, health, and social services have also had demonstrated benefits. In education, for example, students with special health care needs have benefited from greater access (Black et al. 2021). Some students and families have also reported greater flexibility and personalization in education and more effective online learning (Yates et al. 2021). In health and human services that moved to virtual formats, no-show rates decreased as logistical or stigma-related barriers to care were diminished and online appointments were more comfortable for individuals with physical or psychiatric disabilities (Chen et al. 2020). Eliminating commute times also increased the possibility of more frequent but shorter appointments—providing better support to individuals or families needing closer professional support or monitoring, such as those in crisis or undergoing medication changes (Chen et al. 2020).

Questions of equity and access are nonetheless critical. Important opportunities exist to build on what we have learned from the pandemic about the challenges to inclusion inherent in virtual services, including via new funding sources (e.g., public and private insurance carriers’ payments for these services, or additional public funding for virtual public education). In particular, evidence that the benefits of virtual services do not accrue equitably underscores the need for investments in improving access to and affordability of broadband infrastructure, technology, and literacy, to ensure that all people can equitably access jobs, education, healthcare, and socialization that have moved to virtual spaces.

## Centering place in the future

COVID-19 has disrupted usual lifeways, systems, and services, including, significantly, our understandings and uses of “home” and “place.” The challenges it has posed to health and wellbeing have fallen particularly heavily on those in marginalized communities and groups. Yet both people and services have also demonstrated important capacities for flexibility and resilience. The implications and impacts of the pandemic, positive as well as negative, continue to unfold. None of us yet know quite what meeting the demands of even the near-future will entail. Building on the insights available to us, however, we now turn to some (necessarily brief) thoughts on possibilities for a re-envisioning of place, home, and related practices going forward. The COVID-19 pandemic is only one (major) perturbation in what is likely to be an ongoing series of global challenges, given climate change, weather-related and other disasters, and the considerable likelihood of future pandemics. The lessons of this pandemic are thus instructive as a basis for handling the challenges to come, which by their nature are likely to once again have significant impacts on people’s lives and wellbeing in everyday places.

### Opportunities for place-based practices

#### The capacity of place-centered policies, systems, and services to pivot

The relative nimbleness and creativity of policy and practice responses to the challenges of the pandemic are striking. Although this responsiveness came at a price—financially and in terms of workforce demands—service systems nonetheless managed to pivot rapidly to virtual or hybrid service models, often with policy and fiscal support from fast-tracked legislation. Given the complex challenges facing societies globally, we encourage investments in systems and services that sustain this capacity for responsiveness going forward, including flexible funding (and related mindsets) to enable organizations to flex, undertake rapid design work, experiment, test, and (if need be) quickly regroup. These capabilities are as essential in disaster preparedness and response as they have been in the pandemic.

#### The importance of investments in building local connections, capacity, and resilience

From the Brookings Institute (Love 2020) to leading British urbanist Robin Hambleton (2020), a range of commentators has underscored the importance of place as a key locus of intervention post-pandemic. An emphasis on “transformative placemaking” (i.e., innovative, forward-looking investments in places and placemaking that enable collective leadership and problem-solving) is central to these arguments (Love 2020). One key site in these efforts is likely to be “third places”: semi-public settings such as parks, cafes, restaurants, pubs, libraries, farmers’ markets, and gyms that sit between work and home, offering important opportunities for sociability, connection, and imaginative service delivery (Oldenburg 1997). Such spaces hold promise as a key element in the rethinking of urban space that is likely to result from the pandemic, particularly concerning efforts to encourage local spaces that overcome isolation and nurture social connections at manageable (and safe) density levels. Outdoor places hold particular importance in planning for safety from airborne transmissible diseases and in ensuring equity of access for marginalized groups. At the same time, indoor places are also getting much-needed attention, as locations like schools and public housing address long-needed improvements in safety, air quality, and functional design (Lindsley et al. 2021).

#### Re-envisioning home and place

The pandemic has demonstrated that the assumptions of privacy and control over domestic places that prevail in the United States and Canada need to give way to notions of place as shared location and therefore shared care. Realizing this shift will require creative thinking across the inside-outside dialectic, such as finding ways to nurture feelings of at-homeness, connection, and support in settings that span home, school, and neighborhood. The increased use of street spaces and local pocket parks during the pandemic—as classrooms and gyms moved outdoors and cafes offered hampers for picnics—provides just one indicator of how the relationality of local outdoor spaces can be enhanced. Innovative responses to bridging virtual/place binaries will also be needed, such as recent work envisioning digital technologies as platforms for “re-localization” and “re-placing” in newly formed hybrid communities, rather than as primarily supporting trends toward social isolation, disengagement, and displacement (Manzini and Menichinelli 2021, p. 351).

Sustaining these shifts beyond the pandemic will require both conceptual and applied investments, including the development of more expansive guiding frameworks (e.g., Devine-Wright et al. 2020). Partnerships spanning the social, design, planning, and technological professions, in deep collaboration with community stakeholders, are also critical to keeping questions of social care, equity, and solidarity to the fore along with aesthetic, pragmatic, sustainability, and economic priorities. Such broadly transdisciplinary partnerships likewise need to be built into the innovative hyper-local urban strategies such as the 15-Minute City model (Moreno et al. 2021).

### Putting justice in place

Significant paradigm shifts in addressing the socio-spatial inequities brought into sharp relief by the pandemic will not be realized without reckoning with the power structures that dictate regulation and investment in new forms of social care. No amount of re-envisioning will matter for most members of our communities unless it carries across divides of power. People from marginalized backgrounds continue to experience greater disparities in the place-based risks and consequences of the pandemic, economically and epidemiologically. For example, crowding, environmental hazards, and poor ventilation increase risk for contracting or sustaining serious consequences of COVID-19. Many neighborhoods have food, social service, and health care deserts that make resources and care difficult to access, including COVID-19 treatment or prevention. Large numbers of people have no choice but to remain in dangerous homes, neighborhoods, and workplaces. Questions of social justice and equity are thus fundamental to re-envisioning place in the context of health and social policies and services.

## Conclusion

We began this paper by asking how COVID-19 has re-constituted places and people’s relationships with them, and the implications of these changes for social and health services. Our review of emerging research highlights not only the centrality of place in responses to the pandemic and people’s experiences of it, but the complexities entailed in meeting essential human needs and sustaining wellbeing in the context of unprecedented spatial constraints. Our assessments suggest that navigating and responding to these complexities requires not so much new conceptions of place as newly invigorated—and significantly more nuanced and dimensional—discussions around place and its functions. In particular, we see a need to call into question guiding assumptions that frequently reflect uncritical, often overly simplified understandings of people–place relationships. Place holds the potential for so much of what we all long for—satisfaction of our practical and economic needs, peace, comfort, safety, health, connection, and meaning. Yet, in multiple ways across multiple scales, the pandemic has clearly demonstrated that place has the capacity to not only ensure wellbeing but to threaten it. Capitalizing on this moment of re-examination means that scholars, practitioners, and policy makers concerned about the intersections of place, social services, and wellbeing will need to take seriously the ways that people depend on, interact with, and continually (re)create the places they inhabit, across diverse positionalities and power differentials.
